# A Mathematical Framework for Predicting Lifestyles of Viral Pathogens

**DOI:** 10.1007/s11538-020-00730-1

**Published:** 2020-04-29

**Authors:** Alexander Lange

**Affiliations:** 1 Department of Applied Biosciences and Process Engineering, HS Anhalt, Köthen, Germany; 2 Computational Systems Biology, Zuse Institute Berlin, Berlin, Germany

**Keywords:** Infectious disease modeling, Contact networks, Viral fitness, Evolution of viral infections, Infection types, Phylodynamic patterns

## Abstract

Despite being similar in structure, functioning, and size, viral pathogens enjoy very different, usually well-defined ways of life. They occupy their hosts for a few days (influenza), for a few weeks (measles), or even lifelong (HCV), which manifests in acute or chronic infections. The various transmission routes (airborne, via direct physical contact, etc.), degrees of infectiousness (referring to the viral load required for transmission), antigenic variation/immune escape and virulence define further aspects of pathogenic lifestyles. To survive, pathogens must infect new hosts; the success determines their fitness. Infection happens with a certain likelihood during contact of hosts, where contact can also be mediated by vectors. Besides structural aspects of the host-contact network, three parameters appear to be key: the contact rate and the infectiousness during contact, which encode the mode of transmission, and third the immunity of susceptible hosts. On these grounds, what can be said about the reproductive success of viral pathogens? This is the biological question addressed in this paper. The answer extends earlier results of the author and makes explicit connection to another basic work on the evolution of pathogens. A mathematical framework is presented that models intra- and inter-host dynamics in a minimalistic but unified fashion covering a broad spectrum of viral pathogens, including those that cause flu-like infections, childhood diseases, and sexually transmitted infections. These pathogens turn out as local maxima of numerically simulated fitness landscapes. The models involve differential and integral equations, agent-based simulation, networks, and probability.

## Introduction

In view of the many incurable and newly emerging viral infections, such as HIV, HCV, pandemic influenza, dengue, SARS or Ebola, to mention a few, one is interested in knowing more about the ways harmful viruses can exist in the human host population. By employing numerical models, we are trying to learn about their basic reproductive strategies and how these strategies depend on the viral host environment.

Due to the complexity of viral habitats—often located within several host species—and due to the various transmission routes between hosts, which can involve special environmental conditions [e.g., temperature (Handel et al. 2013)], there is no consistent mathematical framework for studying more general virus-related questions. Most of the literature studies particular infections (Murillo et al. 2013; Fraser et al. 2014) and often either focuses on between- (Fraser et al. 2007) or on within-host dynamics (Alizon et al. 2011; Johnson et al. 2012; Handel et al. 2014). However, some articles follow a more general approach, e.g., combine inter- and intra-host dynamics (Coombs et al. 2007; Luciani and Alizon 2009; Pepin et al. 2010), discuss involved challenges (Handel and Rohani 2015; Gog et al. 2015; Lloyd-Smith et al. 2015), or sketch a unified perspective (Grenfell et al. 2004; Lange and Ferguson 2009; Weitz et al. 2019). Two of the last three are of particular interest here, covering the viral phylodynamics of Grenfell et al. (2004) and an epidemiological approach suggested by Lange and Ferguson (2009). As being far from obvious, one would like to know if the two approaches lead to the same conclusions.

Translation between different frameworks is usually not straightforward. Therefore, our first goal aims at establishing interpretation: we want to re-identify concepts from Grenfell et al. (2004) within the framework of Lange and Ferguson (2009). In particular, we try to relate the so-called static patterns of Grenfell et al. (2004) and the infection types of Lange and Ferguson (2009). Besides mathematical structure, a crucial part of any modeling framework is the involved parameters, which we intend to compare and re-identify for the two approaches. We expect that, eventually, this will lead to a similar classification of viruses. Hereby the focus will be on virulent ones, although we do not explicitly vary virulence in our models. Furthermore, we aim to reconstruct the infection types of Lange and Ferguson (2009) by suggesting a minimal set of parameters that allows us to mathematically formulate viral lifestyles and the fitness optimization behind.

As any form of life, the evolutionary success of viruses correlates with their success to reproduce. To take this into account, we study viral replication within and between hosts. Yet, when pursuing the minimalistic approach of Lange and Ferguson (2009), we only follow one particular pathogen at a time, which—determined by its temporal load—spreads in the contact neighborhood of one infected individual. That is, we do not consider multiple infections and thus ignore the interaction between them (Alizon et al. 2013; Gulbudak and Weitz 2019; Clay and Rudolf 2019). This may characterize our approach as rather crude and impose implicit assumptions on the modeled system such as low incidence and a homogeneous host population. Less virulent viruses are excluded by introducing a stopping condition in the within-host model, which will restrict the follow-up period to be less than 2 years.

Following the methods used by Lange and Ferguson (2009), we employ differential- and integral equations, networks, stochastic models, and numerical simulations. Based on the various parameter sets that are involved, we investigate conditions that maximize the reproductive success of the virus, formulated by a version of the basic reproduction number. The maxima are obtained by systematically testing parameter combinations, also at the boundary of the considered parameter regions.

## Background

Before we start, we briefly recall aspects of the two frameworks, Grenfell et al. (2004) and Lange and Ferguson (2009), that are important here.

### The Phylodynamic Framework

**Fig. 1:**
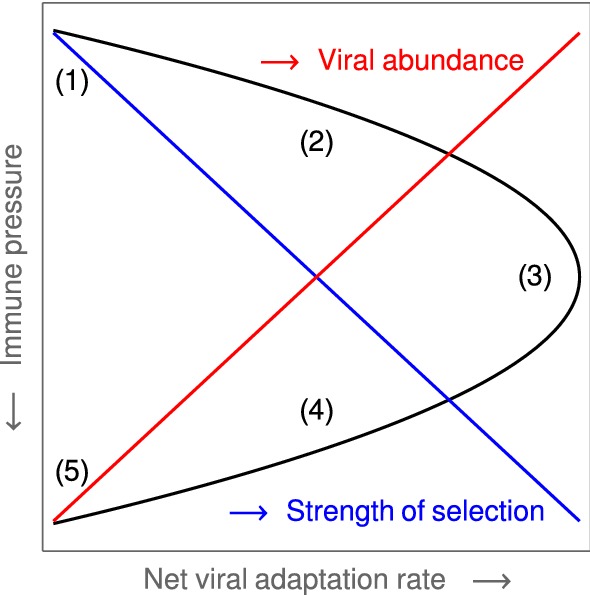
Static patterns. The figure is a $90^\circ$-rotated sketch of Figure 2A in Grenfell et al. (2004). It indicates the locations of the five *static patterns* (lying on a parabola) in the pathogen parameter space of Grenfell et al. (2004), which is formed by the *immune pressure* and the *net viral adaptation rate*. Furthermore, the figure indicates the monotonic behavior of the *strength of selection* (blue) and the *viral abundance* (red) with respect to the immune pressure (*y*-axis) (Color Figure Online)

Analyzing the phylodynamics of viruses, the paper by Grenfell et al. (2004) suggests five so-called *static patterns* to characterize the net adaptation rate of a viral population with respect to the host immunity. Pathogen adaptation is understood as the fixation rate of advantageous mutations in viral epitopes. Based on a simple population genetic model, this rate is shown to increase with the strength of selection for variants that can evade immunity. However, one obtains an inverse relationship between the immune response and the viral population size so that the highest rate of adaptation occurs at an intermediate level of immunity (Fig. 1). The following patterns and RNA-viruses are identified:

1. no effective immune response, no adaptation (HCV in immuno-compromised hosts, influenza A virus immediately after an antigenic shift);
2. low immune pressure, low adaptation (rapidly progressing chronic HCV and HIV);
3. medium immune pressure, high adaptation (antigenic drift in influenza A virus, intra-host HIV infections);
4. high immune pressure, low adaptation (HIV in long-term non-progressive hosts);
5. overwhelming immune pressure, no adaptation (measles and other morbilliviruses).

The paper also discusses how these patterns and corresponding phylogenetic trees emerge based on the intra-host dynamics of the pathogen. For more detail, we refer the reader to the original literature.

### Transmission Mechanisms and Viral Evolution

**Fig. 2:**
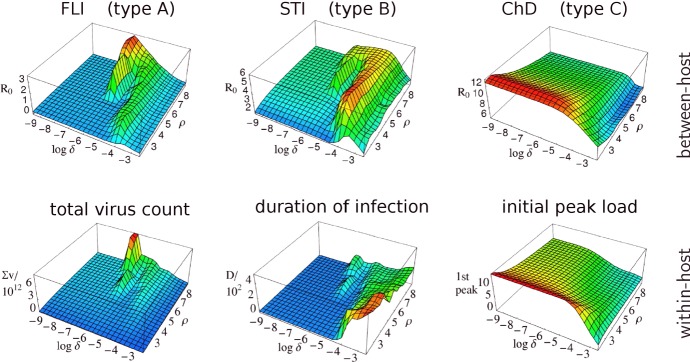
Infection types. This figure is adopted from Figure 3 in Lange and Ferguson (2009). The top row shows the fitness landscapes (due to between-host replication, $$R_0$$) over pathogen space ($$=$$*antigenic variation*$$\delta $$$$\times $$*intra-host replication*$$\rho $$) for flu-like infections (FLI), sexually transmitted infections (STI), and childhood diseases (ChD). The bottom row shows the corresponding between-host characteristics: total virus count ($$\Sigma \,v$$), duration of infection (*D*), and the initial peak load ($$\Sigma \,v\times D$$  for the 1st peak), respectively. The maxima of these surfaces define three evolutionary strategies (or *lifestyles*, as we also refer to them). While having the maxima at the same location in pathogen space, the surfaces of the top and bottom rows are similar too (Color Figure Online)

The work by Grenfell et al. (2004) focuses on the viral population and the host-immune response. Epidemiological aspects such as transmission and inter-host environment are less important in their approach. This is different in the approach by Lange and Ferguson (2009), where infectious diseases are classified into three types (cf. Fig. 2). Even if the classification is based on antigenic variation (being either A: medium, B: high, or C: low), epidemiological aspects such as the host-contact rate and the transmission mode are revealed to be closely related. Each infection type corresponds to a certain range of contact/transmission rates (A: low, B: medium, C: high). Depending on that range, each infection type shows a distinct fitness landscape (between-host reproduction) over pathogen space (Fig. 2, top row). Most interestingly, the infection types correspond to three evolutionary strategies (Fig. 2, bottom row):

1 $$\begin{aligned} {\left\{ \begin{array}{ll} A\\ B\\ C \end{array}\right. } \text {maximizes the}\quad {\left\{ \begin{array}{ll} \text {total viral load,}\\ \text {duration of infection,}\\ \text {initial peak load,} \end{array}\right. } \end{aligned}$$

where, to some extent, the fitness landscapes (top rows) resemble the strategic ones (bottom rows in Fig. 2). The numerical results have been reproduced by Viljoen et al. (2018); differences regarding the conclusions can be pinpointed to modifications of the original method (e.g., the missing stopping condition or the utilization of the Levenberg–Marquardt algorithm, which only finds local extrema and usually not those at the boundary).

## Methods

**Fig. 3:**
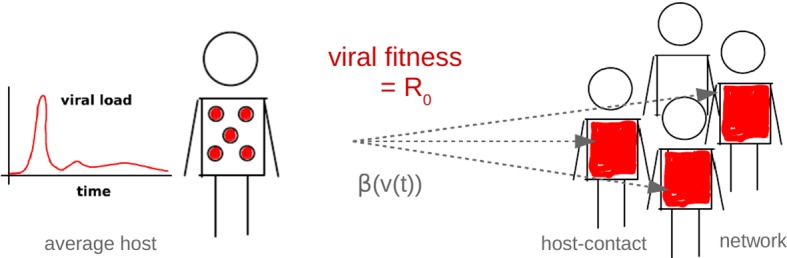
Modeling framework. Systematically, for all viruses represented by our pathogen parameter space, we simulate the within-host evolution and calculate the average load over time *v*(*t*). The load curve is used to define a time-dependent transmission rate, $\beta (v(t))$. Based on this rate, the between-host dynamics is simulated for a totally susceptible host-contact network. The total number of infected individuals then determines the basic reproduction number $R_0$, our model for viral fitness (Color Figure Online)

We study a highly simplified scenario of viral replication that includes intra- and inter-host dynamics (cf. Fig. 3). The link between the two is established by a transmission model, which, following Lange and Ferguson (2009), leads us to quantifying viral fitness in terms of a version of the basic reproduction number $R_0$. Despite well-known limitations of $R_0$ as a fitness measure, referring to the findings of adaptive dynamics (Mylius and Diekmann 1995; Metz et al. 2008; Dieckmann 2002) but also to the definition of $R_0$ (Diekmann et al. 1990; Grassly and Fraser 2006; Li et al. 2011), the proposed $R_0$ will be sufficient for recovering the static patterns of Grenfell et al. (2004) as well as pointing at underlying viral strategies. The intra-host model involves cells for viral replication and an adaptive immune response. Via mutations, viral replication includes a stochastic element. The simulation outcome represents the load of a particular mutable virus in an average host. While, for simplicity, all host individuals are considered equal, our inter-host model does involve structure of a contact/transmission network.

### Viral Fitness Model

In an inter-host context, viral fitness is determined by the success of the virus to reproduce while reaching new hosts. This includes viral reproduction within hosts and transmission to other hosts. Formalized by the viral load, which counts the virions within one host, and the basic reproduction number, which counts new infections in a susceptible host population, these two concepts will provide mathematical expressions that can be utilized to define viral fitness. In contrast to many epidemiological applications that are based entirely on mass-action (Anderson and May 1982), when modeling network structure in the host population, we use the reproduction number in a slightly different way.

In epidemiology, the basic reproduction number $R_0$ measures the fitness of an epidemic (i.e., predicts its survival as long as $R_0>1$). Even if epidemiologists do not use this jargon, an epidemic forms a collective entity of individuals infected with a particular pathogen. Consequently, reproduction is recorded with respect to the disease free equilibrium, imposing a completely susceptible host-population. When studying the fitness of a virus, we impose complete susceptibility only at the beginning of the epidemic. During its course, susceptible numbers in the neighborhood of an infected individual are considered to change. Susceptible hosts form the limited resource that a particular virus—mediated through the contact behavior of hosts—competes for.

The basic reproduction number is defined by the number of secondary infections in a totally susceptible population caused by one initially infected individual and, as employed here, through direct transmission. The initially infected host is supposed to carry the virus to which we intend to assign a fitness value. The secondary infections that are relevant for the viral fitness only represent a subset of individuals that are affected by the epidemic. Initially, the contact neighborhood of the one infective individual only contains susceptibles, $S(0)=N-1$, but, later on, it also contains screened individuals (i.e., non-susceptible individuals that were infected earlier on by secondarily infected individuals).

When modeled by mass-action, the growth of the number of infections resulting from one infective individual, $I(0)=1$, is given by $I'(t)=\beta (t)\,S(t)\,I(0)$. Integration over time then yields $R_0$.

2 $$\begin{aligned} R_0=\int _0^D\beta (t)\,S(t)\,\hbox {d}t. \end{aligned}$$

In practice, one must introduce a cut-off as an upper time limit. This cut-off is modeled by the first entering time, $D=\inf \{t>0\,|\,v(t)\le v_0\}$, capturing the time (referred to as *duration of infection*) when the viral load *v*(*t*) falls below a critical value $v_0$. It is crucial to employ a stopping time here, from a mathematical but also an epidemiological point of view. Namely, we are interested in modeling harmful viruses, which are present at sufficiently high loads, inducing strong immune responses and destroying significant numbers of target cells; we are not interested in learning about viruses that are tolerated by the host at low loads. In our simulations, *D* turned out to be shorter than 2 years. Without a load threshold, as pointed out by Viljoen et al. (2018), an unlimited duration of infection may favor only a single infection type [type B, referred to as milker-like in Viljoen et al. (2018)].

It is important to note that our viral fitness measure (2) coincides with the basic reproduction number as defined in epidemiology only locally. *Local * refers to the environmental parameters (e.g., the size of the contact neighborhood). Formal problems arise when the transmission mode and hence network parameters change. Then one must redefine these parameters and, as a consequence, $R_0$ values might differ drastically [cf. Fig. 5A in Lange and Ferguson (2009)].

### Intra-host Model

**Fig. 4:**
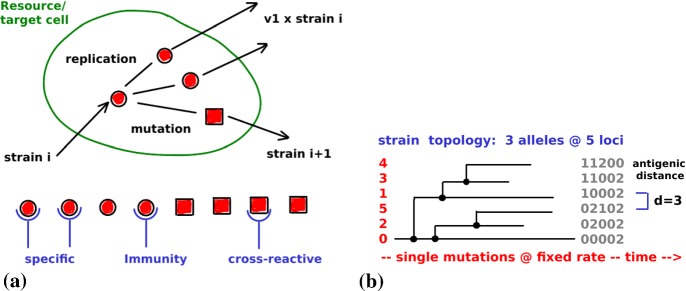
Within-host replication. **a** The replication of viral strain *i* into multiple identical copies (about $\nu _1$) and the mutant strain $i+1$. Before specific immunity develops, there is a cross-reactive immune response from the earlier strain *i*. Cross-reactive immunity is exerted based on the loci-allele structure indicated in (**b**). Its strength depends on the antigenic distance between the involved strains; cf. Eq. (3). The distance is associated with the number of mutations required to transform one strain into the other (Color Figure Online)

For the viral dynamics within the host, we apply one of the simplest compartmental models (Lange and Ferguson 2009) that involves multiple viral strains, adaptive immune responses, and target cells that provide the resource for viral replication; see Fig. 4a. In part, replication is assumed to lead to mutations (governed by a Poisson process of rate $\mu \rho$) and to the creation of novel strains (at frequency $\delta$). The vast majority of the mutations, however, is assumed to be detrimental to the virus. The antigenic appearance of the virus (modeled through a loci-allele structure as illustrated in Fig. 4b) varies between different strains. Mutations are not supposed to change intra-host parameters, except for $\delta ,\rho$. Primarily, immunity is directed toward one specific strain, although it is assumed to provide cross-protection from other antigenically close strains. Mathematically, the immune response (toward strain *i*) is modeled via a function,

3 $$\begin{aligned} y_i(x)=\sum _{k\le n} x_k\cdot \left[ 1-(1-\chi )\,\varrho _{ik}\right] _++\varepsilon >0, \end{aligned}$$

that accumulates all the available amounts $x_k$ of specific immunity weighted by the antigenic distance ($\varrho _{ik}=\#\text { non-coinciding loci of strains } i \text { and }k$; cf. Fig 4b). This function depends on a cross-immunity parameter $\chi \in [0,1]$; in this paper it is supposed to cover innate immunity $\varepsilon$ as well. The bracket denotes the positive part (i.e., $[\,\cdot \,]_+=(|\cdot |+\cdot \,)/2$).

Between mutation events that lead to novel strains, the time evolution of viral loads $v_i$, of specific immunity $x_i$, and of target cells *c* is modeled by a system of asymptotically linear ODEs,

4a $$\begin{aligned} \frac{\text {d}v_i}{\text {d}t}&=(1-\mu )\,\rho \,v_i^+(c) -\sigma \,v_i^-(x), \end{aligned}$$

4b $$\begin{aligned} \frac{\text {d}x_i}{\text {d}t}&=\xi \,(x_0-x_i)+\zeta \,x_i^+(v_i), \end{aligned}$$

4c $$\begin{aligned} \frac{\text {d}c}{\text {d}t}&=\gamma \,(c_0-c)-\rho \,c^-(v). \end{aligned}$$

Novel strains *j*, produced by a Poisson process of rate $\delta \mu \rho$, are introduced by a set of two new equations (e.g., with index *j*) and initial values ($v_j(0)=10$, $x_j(0)=1$).

The response to the virus is based on the following interaction terms (that model)

5a $$\begin{aligned} v_i^+(c)&=v_i\cdot h_{v/\nu _1}(c)&\text {(replication of strain } i \text { depending on the available target cells)}, \end{aligned}$$

5b $$\begin{aligned} v_i^-(x)&=v_i\cdot y_i(x)&\text {(removal of strain }i\text { due to the immune response)}, \end{aligned}$$

5c $$\begin{aligned} x_i^+(v_i)&=x_i\cdot h_\eta (v_i)&\text {(activation of specific immunity to strain } i), \end{aligned}$$

5d $$\begin{aligned} c^-(v)&=c\cdot h_{c}(v/\nu _1)&\text {(target cell depletion due to infection)}; \end{aligned}$$

the involved rates are listed in Table 1. Hill functions $h_a(b)=\frac{b}{a+b}\in [0,1]$ are employed to scale the virus production according to the available target cells and to implement a load-dependent immune response. Target cell depletion is derived entirely from virus production, $c^-=\frac{1}{\nu _1}\sum _iv_i^+$. To further illustrate the resulting interactions, we point out when they behave linearly,

6a $$\begin{aligned} v_i^+(c)&=v_i&\text {if}\quad c\gg v/\nu _1\quad \text {(target cell number is large)}, \end{aligned}$$

6b $$\begin{aligned} x_i^+(v_i)&=x_i&\text {if}\quad v_i\gg \eta \quad \text {(strain-specific viral load is high)}, \end{aligned}$$

6c $$\begin{aligned} c^-(v)&=c&\text {if}\quad v\gg \nu _1c\quad \text {(viral load is high)}. \end{aligned}$$

Under opposite conditions, each of these terms vanishes. In particular, $v_i^+(c)=0$ if $c\ll v/\nu _1$, which reflects saturation effects caused by the limited number of target cells. In the virus-free equilibrium, all the interaction terms vanish and the system of ODE decouples: $v_i=0$, $x_i=x_0$, $c=c_0$.

### Transmission Dynamics

According to our fitness definition, we need to study viral transmission between hosts. As motivated in Sect. 3.1, we assume that the rate of transmission depends on the viral load *v* of the transmitting (average) host. A simple model is given by an exponential law [cf. Fig. 1 in Lange and Ferguson (2009)],

7 $$\begin{aligned} \beta =\widehat{\beta }\cdot (1-e^{-\alpha \,v}) \end{aligned}$$

where $\alpha$ represents a load-dependent infectiousness parameter and $\widehat{\beta }$ the load-saturated transmissibility (transmission rate per capita). This coefficient,

8 $$\begin{aligned} \widehat{\beta }=\frac{\kappa \,\lambda }{N}, \end{aligned}$$

which is taken with respect to a reference population, is formed by the product of the contact rate $\kappa$ and the likelihood $\lambda$ of transmission per contact over the average number *N* of individuals in the contact neighborhood of a single host. The parameters $\alpha$, $\widehat{\beta }$, and *N* encode the mode of transmission. Typical values are given in Table 2 and Fig. 6.

As a consequence of within-host dynamics and time-dependent viral load *v*(*t*), the transmission rate is also a function of time, $\beta (t)$. Its initial value corresponds to the viral load at the time of infection, $t=0$.

**Table 1:** Fixed parameters

Symbol	Parameter
$c_0$ ($10^8$)	Initial/max resource
$v_0$ (10)	Initial/min viral load
$x_0$ (1)	Initial/min immunity
$\widehat{\alpha }$ ($10^{-5}$)	Upper infectiousness bound
$\gamma$ (1)	Replenishment of resource
$\varepsilon$ ($0.25/\sigma$)	Innate immunity
$\zeta$ (0.8)	Growth of immunity
$\eta$ ($10^3$)	Saturation of immunity
$\mu$ (0.1)	Mutation rate
$\nu _1$ ($10^3$)	Virions per resource unit
$\xi$ (0.3)	Decline of immunity
$\sigma$ ($10^{-3}$)	Clearance due to immunity
$\varphi$ (0.25)	Cliquishness
$\chi$ (0.4)	Cross-immunity

Values we used are given in brackets. Time units are always days

**Table 2:** Transmission parameters

Infection	type	$\lg \,(\widehat{\beta }\times \text {days})$	*N*	$R_0$	$T/\text {days}$
ChD	C	$-\,1$	15	15	10
STI	B	$-\,2$	3	6	200
FLI	A	$-\,3$	400	2	5

Exemplary values of the transmissibility $\widehat{\beta }$ for three infection types (cf. Fig. 2), estimated in accordance with values for a single host’s neighborhood size *N*, the basic reproduction number $R_0=T\times N\times \widehat{\beta }$, and the mean infectious period *T*. Note that these values are all ballpark figures because, even for the same infection, $R_0$ is known to vary hugely (Guerra et al. 2017; Delamater et al. 2019)

### Host Network

The viral dynamics between hosts is modeled most realistically on a network, where potential hosts represent the nodes linked to each other via potential contacts. A particular fraction of contacts ($\lambda$, specific to the infection) transmits the virus from one to another host. To quantify the reproductive fitness of the virus, we study the transmission network only for the contact neighborhood of one initially infected host. For this neighborhood, consisting entirely of susceptibles at the beginning, we determine the changing number of susceptibles over time and calculate the basic reproduction number (2), defined similarly to an effective reproductive number suggested for time-depending transmission rates and systematically varying numbers of susceptibles (Grassly and Fraser 2006). We do not explicitly consider intermediate hosts or vectors here, but neither we exclude them; mass-action can provide an effective description (Lange 2016).

**Fig. 5:**
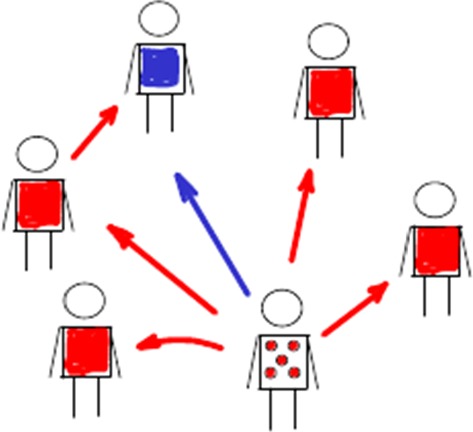
Screening effect. The Sketch illustrates the reduced number ($4<5$) of susceptibles (red) in the contact neighborhood of one infected individual (red dots), caused by one secondary infection in the host network ($\varphi \ne 0$). The *screened * individual (blue) cannot be infected by the initially infected individual anymore. This reduces the basic reproductive number in comparison with an idealistic network-free scenario ($\varphi =0$) (Color Figure Online)

Different from a simple mass-action model, the mathematical formalism describing a network incorporates a cliquishness parameter $\varphi$, which quantifies the number of contacts between members of the considered network-neighborhood. Including a network structure is crucial. Network contacts help spreading the virus through the neighborhood and, as a consequence, effectively lower the number of susceptibles in that neighborhood. Being similar to the screening of charges in a solvent (Debye and Hückel 1923), we refer to this phenomenon as screening effect (cf. Fig. 5); in ecology the effect is also known as self-shading (Messinger and Ostling 2013). The phenomenon cannot be modeled via a modified mass-action coupling alone, yet screening seems to be necessary for obtaining type C infections [cf. Fig. 4 in Lange and Ferguson (2009)].

In the contact neighborhood of the initially infected host, the spread of the virus can be described in terms of two compartments, representing real-valued numbers (i.e., normalized densities) of susceptible *S* and infective individuals *I*. The generation of infected individuals (at time *t*) is given by

9a $$\begin{aligned} I'(t)&=S(t)\,\beta (t)+\varphi ~S(t)\int _0^t\hbox {d}\tau _1\,\beta (t-\tau _1)\,I'(\tau _1) \end{aligned}$$

9b $$\begin{aligned}&\quad +\varphi ^2\,S(t)\int _0^{t}\hbox {d}\tau _2\,\beta (t-\tau _2) \,S(\tau _2)\int _0^{\tau _2}\hbox {d}\tau _1\,\beta (\tau _2-\tau _1)\,I'(\tau _1)+\cdots , \end{aligned}$$

where the listed terms model transmissions from the initial host, secondary hosts (infected by the initial host at time $\tau _1$), tertiary hosts (infected by secondary hosts at time $\tau _2$), etc. All these terms represent mass-action coupling, and stochastic effects are ignored here. Transmissions from secondary hosts are weighted by the network parameter $\varphi$, tertiary hosts by its square $\varphi ^2$, etc. The involved convolution products,

10 $$\begin{aligned} (\beta *I')(s)=\int _0^s\hbox {d}\tau \,\beta (s-\tau )\,I'(\tau )=\int _0^s\beta (s-\tau )\,\hbox {d}I(\tau ), \end{aligned}$$

provide load-weighted transmission rates (at time *s*, originating from new infections before *s*). According to the mass-action law, these terms are multiplied by the numbers of susceptibles *S*(*s*) in Eq. (9).

To obtain an equation that only involves susceptibles, we replace $I'$ by $-S'$ based on the assumption that the size of the contact neighborhood of the initially infected host does not change over time,

11 $$\begin{aligned} N'=S'+I'=0. \end{aligned}$$

The substitution is applied to Eq. (9) and, to save computation time, only secondary hosts (9a) are considered. The resulting equation,

12 $$\begin{aligned} S'=-\left( \beta -\varphi \,\beta *S'\right) S, \end{aligned}$$

which models the time evolution of susceptibles in the contact neighborhood of the initially infected, is solved numerically starting with $S(0)=N-1$. The resulting function, *S*(*t*), is then used to calculate the basic reproduction number (2).

There is no need to introduce further compartments. Recovered individuals, for example, are modeled by infectives with low viral load. However, one may include replacement of individuals in the contact neighborhood. Its influence on possible infection types has been studied by Lange and Ferguson (2009) (Fig. 4).

Note that individuals usually live in various contact networks at the same time. This explains why, even if employing a viral fitness measure, an adult infected with an STI can be infected with flu at the same time. In our model, different networks and corresponding infections are treated separately, although they could coexist within one host.

### On the Choice of the Viral Fitness Model

In modern approaches, evolution is studied as a game of invasion requiring a winning trait to represent an evolutionary stable strategy (Smith and Price 1973). When looking at viral evolution from such a perspective, the concept of fitness seems questionable and sometimes even obsolete (Gyllenberg and Service 2011). In adaptive dynamics (Geritz et al. 1998), for instance, one evaluates the success of an invading viral strain (with trait $\theta =\theta _\text {inv}$) in replacing a resident strain based on the reproduction of the new strain at equilibrium densities of the resident strain ($S=S_\text {res}$, $I=I_\text {res}$). Reproduction of the resident strain is given by $R(S=S_\text {res},I=I_\text {res},\theta =\theta _\text {res})=1$, which means that invasion is successful whenever $R(S=S_\text {res},I=I_\text {res},\theta =\theta _\text {inv})>1$.

Unfortunately, for the questions we like to answer, there are conceptual challenges inherent in adaptive dynamics. Those are the proposed equilibrium and the assumed knowledge of the dynamical system. Consider dengue, for example, where one observes circulating strains and no static equilibrium. The precise dynamics is not known either (Lange 2016), and dengue is just one particular infection in a large set of infections that we would like to include. In fact, we do not know how to tackle invasion problems in such a general setting, where one would have to consider several invasions based on unknown dynamics and possibly not even at equilibrium. For now, we can only test which set of strains reproduces best in a given environment. We propose that network effects and the initial period after infection are of particular importance (Georgieva et al. 2019), leading to a classification similar to the one by Grenfell et al. (2004). However, even if nature agrees with our predictions relying on basic reproduction, it remains a scientific task to trace back invasion histories.

Under certain conditions, the concept of maximizing $R_0$ leads to the same conclusions as adaptive dynamics (Cortez 2013). For usual SIR-compartment models, the basic reproductive number $R_0(\theta )$ depending on a single trait parameter $\theta$ has been shown to provide a fitness measure if reproduction at equilibrium $R(\bar{S},\bar{I},\theta )$ can be factorized into $R_0(\theta )$ and a function $g(\bar{S},\bar{I})$ that exclusively depends on the equilibrium values $\bar{S},\bar{I}$ (and not on $\theta$), i.e., if $R(\bar{S},\bar{I})=g(\bar{S},\bar{I})\,R_0(\theta )$. The inequalities mentioned above show that $R_0(\theta _\text {inv})>R_0(\theta _\text {res})$ implies $R(S_\text {res},I_\text {res},\theta _\text {inv})>R(S_\text {res},I_\text {res},\theta _\text {res})=1$.

This idea appears to be applicable to our network model as well. Our network is defined locally employing SI-dynamics, as explained in the previous section, with parameters characterizing the neighborhood of one infected individual. Underlying this approach, we assume that local parameters (e.g., the neighborhood size *N*) can be scaled up to the whole population (given by $\widehat{N}=N/\varphi$, approximately), where each infected individual in the population experiences the same dynamics as the one studied in its neighborhood. (For topological reasons, the size of the whole population is inversely related to the cliquishness parameter $\varphi$.)

For the reproduction at equilibrium, $R(\bar{S},\bar{I},\theta )=1$, one will always find a number $\bar{S}$ depending on the trait $\theta$, for which $g(\bar{S},\bar{I})=1/R_0(\theta )=\bar{S}/\widehat{N}=\varphi \bar{S}/N$. In analogy to usual SIR-compartment models, $\bar{S}$ would define an estimate of the equilibrium number of susceptibles in the whole population and $\varphi \bar{S}$ an estimate for the corresponding number in the neighborhood. Note that the susceptibles in the neighborhood have been quantified by the network topology, but the expression is plausible with respect to the network dynamics as well. Namely, to keep Eqs. (9) and (12) invariant, *S* needs to be scaled with the inverse of $\varphi$, i.e., the product $\varphi \bar{S}$ is likely a constant and, as required by Cortez (2013), the function *g* does not explicitly involve the trait $\theta$.

### Fitness Maxima

In our setting, the basic reproduction number as defined in (2) is assumed to encode viral fitness. It is evaluated for two sets of parameters (two each), $R_0(\widehat{\beta },\alpha ;\delta ,\rho )$, referred to as *pathogen space*$(\delta ,\rho )$ and *transmission space*$(\widehat{\beta },\alpha )$. These spaces are supposed to capture different *types* of viral pathogens.

**Fig. 6:**
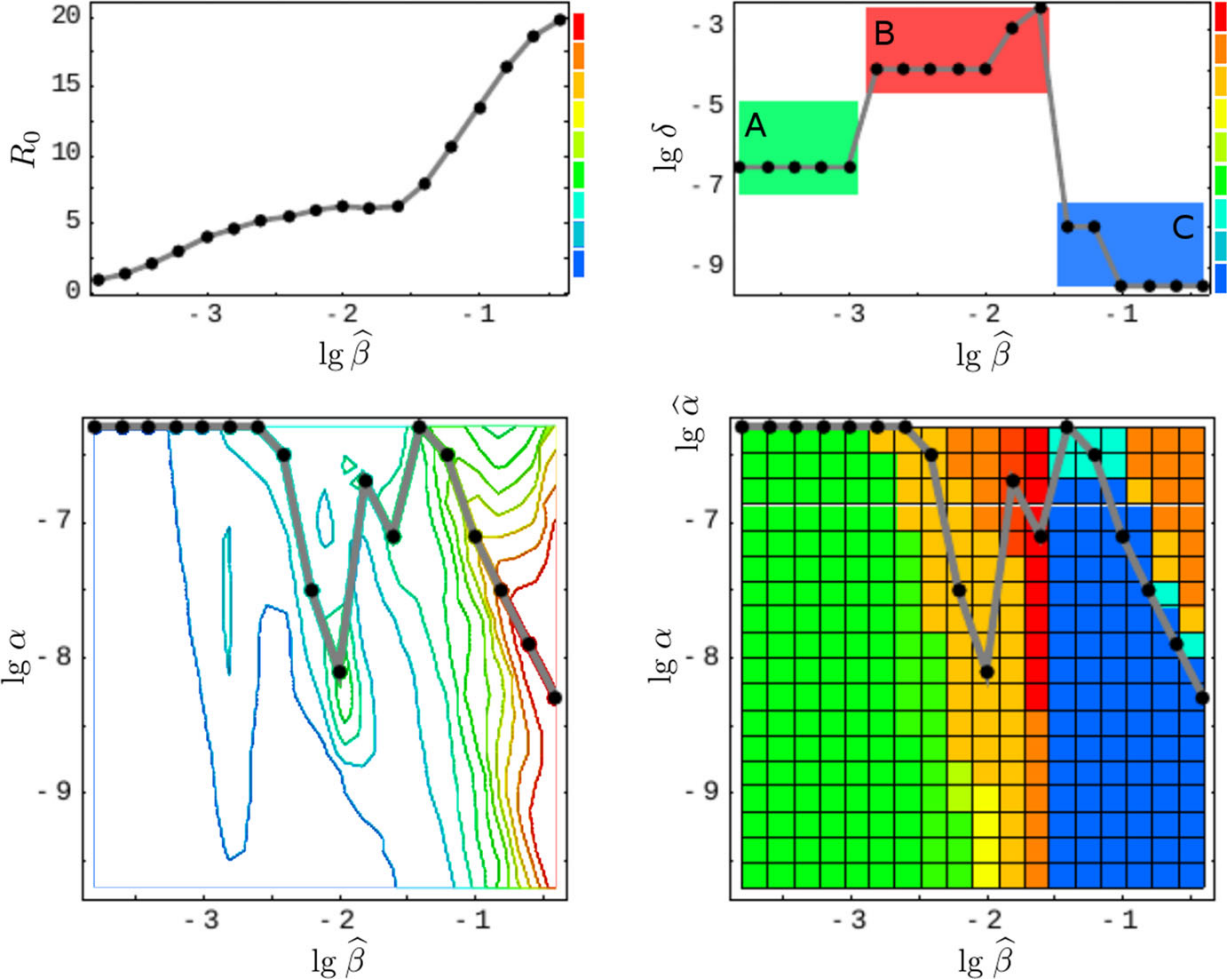
Fitness and antigenic variation. The figure illustrates the definition of the three infection types (A, B, C) based on antigenic variation (medium, high, low) and maximal fitness. The lower left-hand side panel shows the fitness landscape on transmission space, $\max _{\delta ,\rho }\,R_0(\widehat{\beta },\alpha ;\delta ,\rho )$. The corresponding top panel indicates the fitness maxima $\widehat{R}_0(\widehat{\beta })$ for the simulated transmissibilities $\widehat{\beta }$ (black dots). The lower right-hand side panel shows the antigenic variation ($\lg \,\delta$) over transmission space $(\widehat{\beta },\alpha )$. Here, the gray curve $\widehat{\delta }(\widehat{\beta })$ selects the $\delta$-values that correspond to fitness maxima. These $\delta$-values are shown in the corresponding top panel; they suggest a three-type classification (Color Figure Online)

To determine the types that we assume are favored by evolution, we search for parameter values,

13 $$\begin{aligned} \widehat{\delta }(\widehat{\beta })&=\underset{\delta }{\text {arg max}} \left( \max _{\alpha \le \widehat{\alpha },\rho }\,R_0(\widehat{\beta },\alpha ;\delta ,\rho )\right) , \end{aligned}$$

as indicated for the antigenic variation $\delta$ (cf. Fig. 6), that maximize viral fitness,

14 $$\begin{aligned} \widehat{R}_0(\widehat{\beta })&=\max _{\alpha \le \widehat{\alpha }}\,\max _{\delta ,\rho }\, R_0(\widehat{\beta },\alpha ;\delta ,\rho ). \end{aligned}$$

The antigenic variation is of particular importance; It offers a natural classification leading to three infection types (referred to as A,B,C; cf. Fig. 6).

Despite the many trait parameters the fitness function is optimized for, effectively there is only one viral trait required to be imported from the intra-host model (cf. Sect. 3.5); Fig. 6 suggests that $\theta =\lg \,\alpha$. Testing maxima for the environmental parameter $\widehat{\beta }$ appears to be sufficiently general as well . The cliquishness parameter $\varphi$, for example, as being another environmental parameter, can be expressed in (12) by the neighborhood size *N*, to which it is inversely related, $\varphi \propto 1/N$, approximately. (This follows from the fact that simultaneous scaling of *S* and $\varphi$ keeps Eq. (12) invariant.) Further parameters, such as cross-immunity $\chi$ and the infectiousness bound $\widehat{\alpha }$ (both encoding viral traits), represent generic scenarios for a wide range of values. They are kept fixed when deriving our first result, the static patterns. Their influence on the pathogenic lifestyle is investigated afterwards, forming our second result.

## Results

Applying the model outlined above, one can straightforwardly reconstruct the static patterns of Grenfell et al. (2004). Furthermore, one can identify three parameters that—when adjusted appropriately—lead to the three infection types introduced by Lange and Ferguson (2009). This is demonstrated in the following two subsections.

### Reconstruction of the Static Patterns

We assume that the pathogen space of Grenfell et al. (2004) (cf. Sect.2.1) can be identified with ours via the following two correspondences,

15a $$\begin{aligned} \text {1 / immune pressure}~&\sim ~\text {intra-host reproduction, }\rho , \end{aligned}$$

15b $$\begin{aligned} \text {net viral adaptation rate}~&\sim ~\text {antigenic variation, }\delta , \end{aligned}$$

where “$\sim$” encodes positive correlation. Our first parameter, the intra-host reproduction, defines the reaction of the immune system to the virus, whereas our second parameter, the antigenic variation, already coincides with the one utilized by Grenfell et al. By maximizing the basic reproduction number (Eq. (2)) over these two parameters, and keeping all other parameters fixed,^1^ we obtain a $\widehat{\beta }$-depending curve that represents maximal values of viral fitness in pathogen space,

16 $$\begin{aligned} \widehat{\beta }\mapsto \underset{(\delta ,\rho )}{\text {arg max}}~\widehat{R}_0(\widehat{\beta }). \end{aligned}$$

**Fig. 7:**
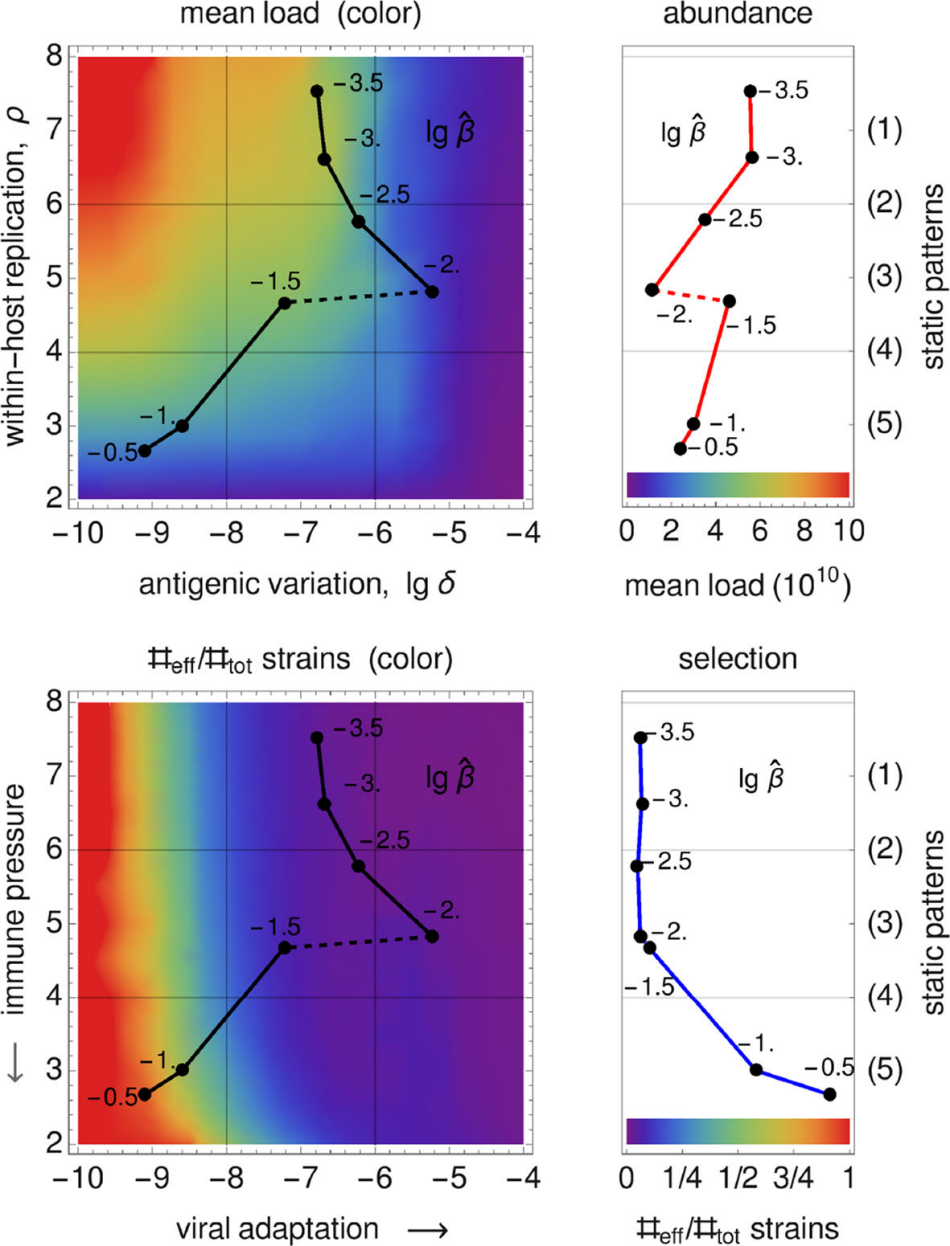
Reconstructed static patterns. The top panels show the mean viral load [called abundance in Grenfell et al. (2004)] over the pathogen space (left) and the fitness maximum over transmissibility (right). The bottom panels show the ratio of effective to total strain numbers [representing the strength of selection in Grenfell et al. (2004)] over the pathogen space (denoted as in Grenfell et al. (2004); left) and the fitness maximum over transmissibility (right). In all four diagrams, the black data points (produced by numerical simulation) coincide. In comparison with Grenfell et al. (2004), five static patterns are identified with particular (ranges of) transmissibility (top and button, right) (Color Figure Online)

This curve (black, in left-hand side panels of Fig. 7) resembles the parabola of Grenfell et al. (2004) (Fig. 1), which defines five static patterns (cf. the right-hand side of Fig. 7). We therefore hypothesize that the five patterns (numbered $1,\dots ,5$) are positively correlated with the transmissibility $\widehat{\beta }$ (cf. left-hand side panels in Fig. 7). In Grenfell et al. (2004), the five patterns have not been associated with inter-host concepts or a particular parameter. Within our framework, the transmissibility $\widehat{\beta }$ offers a natural scale for labeling these patterns. By changing the value of $\widehat{\beta }$, one can shift between patterns.

Furthermore, we are able to reconstruct the *viral abundance* and the *strength of selection* over the range of the static patterns (or, equivalently, the immune pressure; cf. right-hand side panels in Fig. 7). Here the following correspondences are employed,

17a $$\begin{aligned} \text {viral abundance}~&\sim ~\text {mean viral load}~\, (=\bar{v}), \end{aligned}$$

17b $$\begin{aligned} \text {strength of selection}~&\sim ~\text {ratio of effective to total number of strains}~\,(=\#_\text {eff}/\#_\text {tot}) , \end{aligned}$$

where the effective number of strains is associated with load-weighted strain-frequencies, $\#_\text {eff}=\sum _i\bar{v}_i\#_i$, and $\#_\text {tot}=\sum _i\#_i$. For the viral abundance, we obtain a jump between the patterns 3 and 4 (or, equivalently, between $\lg \widehat{\beta }=-2$ and $-1.5$, as indicated by a dotted line in the top left panel of Fig. 7). This discontinuity is visible as well in the maximized fitness curve on the left-hand side panels (indicated by a dotted line again).

**Fig. 8:**
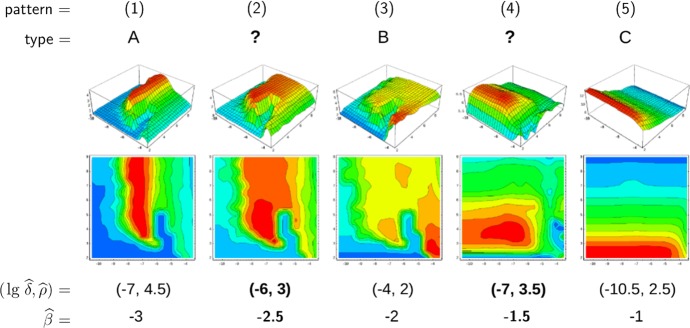
Static pattern versus infection types. For five transmissibilities $\widehat{\beta }$, fitness landscapes $R_0(\delta ,\rho )$ are plotted over pathogen space (3D and via contour) and associated with static patterns and infection types. For two of the transmissibilities (bold), the infection type is not clearly differentiated (between A–B and B–C, marked by “?”). They likely correspond to the static patterns (2) and (4) (Color Figure Online)

To associate the five static patterns and the three infection types in a more conceivable way, we have re-computed the fitness landscapes over pathogen space (Fig. 2, top row) for two more transmissibilities (Fig. 8). Those then correspond to the two remaining static patters, even if it turns out to be difficult to associate these extra landscapes with exactly one of our three infection types. Nevertheless, the transmissibility $\widehat{\beta }$ is seen again to be a natural parameter here.

### Natural Parameter Space

**Fig. 9:**
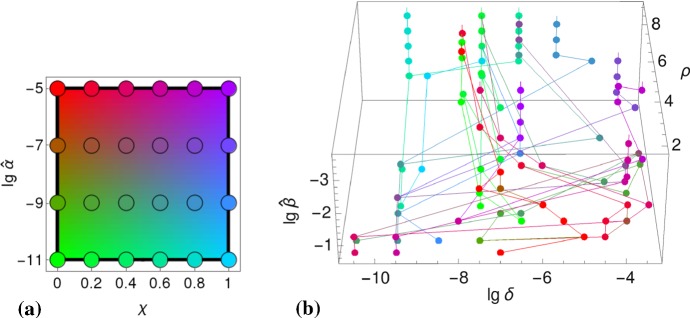
Parameter space. **a** Hue values ($\text {red}\sim \widehat{\alpha }$, $\text {green}\sim 1/\widehat{\alpha }$, $\text {blue}\sim \chi$) that uniquely depend on cross-immunity $\chi$ and the infectiousness bound $\widehat{\alpha }$. **b** The mapping (18) from parameter- to pathogen space; points of the same color—representing the same parameter values $(\chi ,\widehat{\alpha })$—are connected by a thin line (Color Figure Online)

In addition to the transmissibility $\widehat{\beta }$, it is beneficial to also examine the dependence of the viral fitness on cross-immunity $\chi$ and on the infectiousness bound $\widehat{\alpha }$. Therefore we study the mapping

18a $$\begin{aligned} (\chi ,\widehat{\alpha })\mapsto \left( \widehat{\delta }(\chi ,\widehat{\alpha };\widehat{\beta }), \widehat{\rho }(\chi ,\widehat{\alpha };\widehat{\beta });\widehat{\beta }\right) , \end{aligned}$$

illustrated in Fig. 9b, which assigns values of the two parameters $(\chi ,\widehat{\alpha })$—encoded by color (Fig. 9a)—to points in pathogen space that maximize $R_0$,

18b $$\begin{aligned} \big (\widehat{\delta },\widehat{\rho }\big )(\chi ,\widehat{\alpha }; \widehat{\beta })&=\underset{(\delta ,\rho )}{\text {arg max}}~\widehat{R}_0(\chi ,\widehat{\alpha };\widehat{\beta }), \end{aligned}$$

where $\widehat{R}_0(\chi ,\widehat{\alpha };\widehat{\beta })=\max _{\alpha \le \widehat{\alpha }}\,\max _{\delta ,\rho }\,R_0(\chi ,\widehat{\alpha };\widehat{\beta },\alpha ;\delta ,\rho )$. The dependence on the transmissibility $\widehat{\beta }$ is captured by a third dimension, erected over pathogen space $(\delta ,\rho )$.

**Fig. 10:**
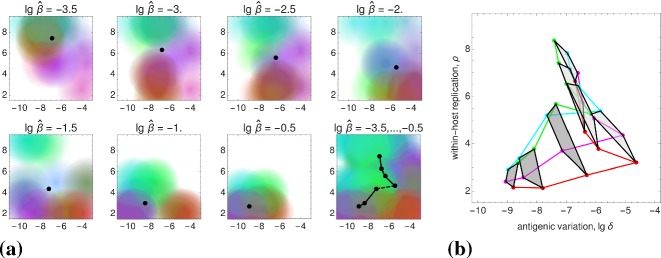
Extrapolation and extremal parameter pairs. **a** The eight panels show the fitness maxima in pathogen space for seven transmissibilities $\widehat{\beta }$ and a cumulative combination of them; the colors uniquely represent parameter pairs $(\chi ,\widehat{\alpha })$ as defined in Fig. 9a. The average $(\widehat{\delta },\widehat{\rho })$-values—taken over the $6\times 4$ parameter pairs $(\chi ,\widehat{\alpha })$—are indicated by black dots; in the cumulative panel, they are connected by black lines. **b** Intensity-weighted average locations of the four extreme parameter pairs $(\chi ,\widehat{\alpha })$ are shown in pathogen space (red, green, violet, cyan in Fig. 9a). Each of the seven quadrilaterals corresponds to one transmissibility ($\lg \,\widehat{\beta }=-3.5,\dots ,-0.5$). The quadrilaterals change their orientation about halfway, when the fitness maxima over $\widehat{\beta }$ show a discontinuity (cf. Fig. 7) (Color Figure Online)

Numerical simulations for our (relatively large) parameter space, which cover the within-host dynamics and the transmission network, are hugely time-consuming. They restrict the parameter pairs $(\chi ,\widehat{\alpha })$—feasible to consider—to be a small number ($=6\times 4$).^2^ Instead of enlarging this number by increasing the computation power/time, we decided to proceed by locally extrapolating the simulation results. That is, we blur the image points of the mapping (18) by “enlarging” these points, so that they become colored circles. At the same time we decrease the intensity of their unique color toward outer radii. As a consequence, colors of nearby circles mix according to their red-green-blue content, and we obtain colored patches in pathogen space where the color content corresponds to a unique $(\chi ,\widehat{\alpha })$-parameter combination. The result of that extrapolation is shown in Fig. 10a.

Complementing the extrapolation, we examine the most extreme $(\chi ,\widehat{\alpha })$-parameter combinations, the corners in Fig. 9a. Here one makes an interesting observation; see Fig. 10b. The discontinuity between the patterns 3 and 4 (cf. Fig. 7) results in a change of orientation:

19a $$\begin{aligned}&\text {for the patterns}~ {\left\{ \begin{array}{ll}1,2,3\\ 4,5\end{array}\right. },~ \text {which correspond to}~ {\left\{ \begin{array}{ll}\text {low}\\ \text {high}\end{array}\right. } \text {transmissibility }\widehat{\beta }, \end{aligned}$$

19b $$\begin{aligned}&\text {high values of cross-immunity }\chi \text { lie at}~ {\left\{ \begin{array}{ll}(\text {high},\text {high})\\ (\text {low},\text {low})\end{array}\right. } \text {values of }(\delta ,\rho ). \end{aligned}$$

In contrast, the values of the infectiousness bound $\widehat{\alpha }$ that maximize viral fitness do not jump in pathogen space: high values of $\widehat{\alpha }$ always lie at $(\text {high},\text {low})$ values of $(\delta ,\rho )$.

**Fig. 11:**
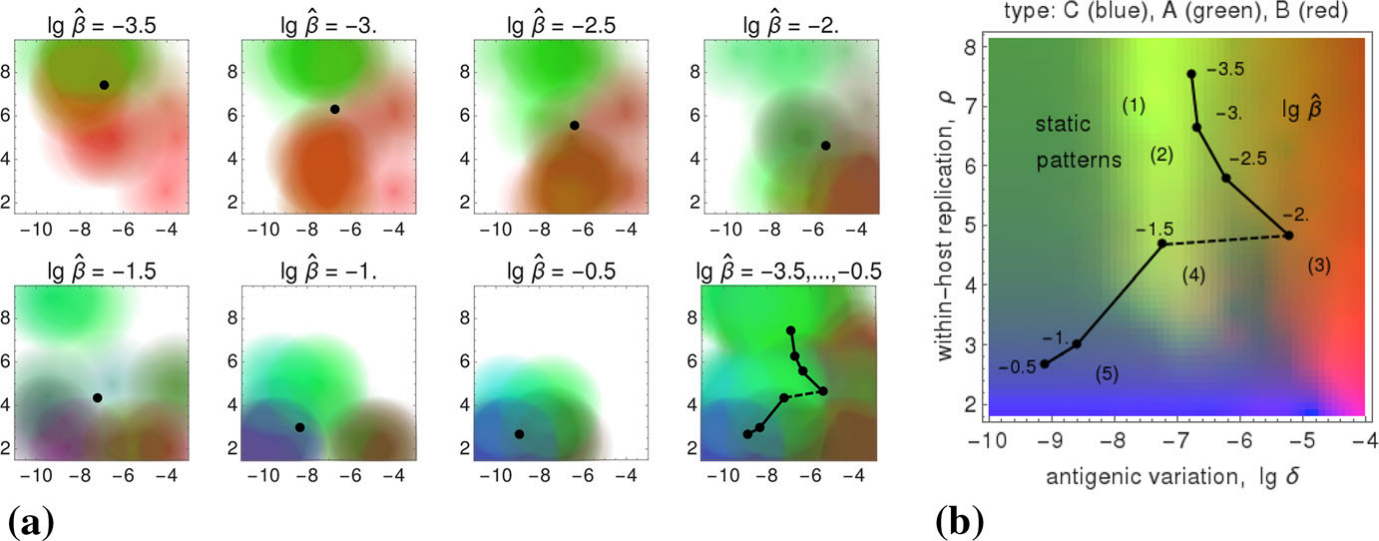
Infection type reconstruction. **a** The eight panels show the fitness maxima in pathogen space for seven transmissibilities $\widehat{\beta }$ and a cumulative combination of them; the colors uniquely represent parameter pairs $(\chi ,\widehat{\alpha })$ as defined by the correspondences (20). The average $(\widehat{\delta },\widehat{\rho })$-values (taken over all colors) are indicated by black dots, which in the cumulative panel are connected by lines. **b** The infection types A, B, C (colored blue, red, green, resp.) are located in pathogen space, as well as the static patterns (1,...,5) and the transmissibility $\widehat{\beta }$; the resulting color distribution is approximated well by the cumulative diagram “$\lg \,\widehat{\beta }=-3.5,\ldots ,-0.5$” in (**a**) (Color Figure Online)

By linear combinations of the parameter content (illustrated by the red-green-blue mixing of colors in Figs. 10, 11), the results above can be used to roughly reconstruct the infection types of Lange and Ferguson (2009) in terms of three modeling parameters, $\chi ,\widehat{\alpha },\widehat{\beta }$. These parameters (i.e., their combined values) are supposed to represent *lifestyles*. Based on the color code for antigenic variation (cf. Fig. 6), we propose the following simple dependencies,

20a $$\begin{aligned} \text {fitness of type B}&\,\sim \,\widehat{\alpha }, \end{aligned}$$

20b $$\begin{aligned} \text {fitness of type A}&\,\sim \,1/(\widehat{\alpha }\cdot \widehat{\beta }), \end{aligned}$$

20c $$\begin{aligned} \text {fitness of type C}&\,\sim \,\widehat{\beta }\cdot \chi , \end{aligned}$$

where $\chi ,\widehat{\alpha }$ contribute hue values as seen in Fig. 10a and defined in Fig. 9a, and where $\widehat{\beta }$ provides an intensity weight in accordance with (19). The resulting color distribution, i.e., the “mixture” of lifestyles over pathogen space, is shown in Fig. 11; the similarity of the color content in (a) and (b)—corresponding to the right- and left-hand side expressions in (20), respectively—is clearly visible.

It is not difficult to explain how these relations, Eqs. (20), have been obtained. The infectiousness bound $\widehat{\alpha }$ (occurring only in Eqs. 20a and b) selects between the types A and B: if low (i.e., if high loads are required for transmission), type A (i.e., FLI) is favored; if high (i.e., if low viral loads are sufficient), type B (i.e., STI) is favored. According to (19), both these types are favored by rather low transmissibility $\widehat{\beta }$. Cross-immunity $\chi$ (scaled blue; cf. Fig. 9a) favors two patches in pathogen space (cf. Fig. 10a). The one with high transmissibility $\widehat{\beta }$ corresponds to type C (i.e., ChD), the other we do not really know. It might represent vector-born infections (Lange and Ferguson 2009), but it is not type C. Fortunately, this does not matter as in Fig. 11a the blue color is switched off at low transmissibility $\widehat{\beta }$ (cf. Eq. 20c). If $\widehat{\alpha },\chi$ are kept fixed, as in Sect. 4.1, only the transmissibility selects the infection type in (20): A for low-, C for high-, and hence, B for medium $\widehat{\beta }$-values.

## Discussion

Summarizing these last results, we have proposed a mathematical framework equipped with various sets of parameters that allows for predicting different types and lifestyles of viral pathogens. Types refer to the antigenic variation, lifestyles to the evolutionary strategy and corresponding parameter values that maximize fitness (cf. Figs. 2, 8). The parameter sets—forming so-called pathogen- and transmission spaces—cover intra- and inter-host dynamics, including a simple host-contact/transmission network. Three parameters are necessary for the reconstruction of the observed types/lifestyles: the infectiousness bound $\widehat{\alpha }$ and the transmissibility $\widehat{\beta }$, which restrict the possible modes of transmission, and the cross-immunity parameter $\chi$. The relations (20) establish fitness definitions (Fig. 11a) for the three infection types of Lange and Ferguson (2009) (cf. Fig. 11b). These relations were obtained by visual inspection (comparison of Fig. 11a, b); they could be refined by using statistical tools.

Furthermore, referring to the results presented earlier in the paper, we have given an epidemiological interpretation of the static patterns in the phylodynamic theory of Grenfell et al. (2004). We claim that the transmissibility $\widehat{\beta }$ is of particular importance. By only adjusting its value, transitions between the five static patterns and, correspondingly, the three infection types are possible. Explicitly, this means that the transmissibility and, more general, the contact behavior determine the lifestyle of the considered pathogens. The transmissibility $\widehat{\beta }$ offers a natural (epidemiological) parameterization of the hand-sketched parabola by Grenfell et al. The similarity of the functional dependencies expressed by that parabola (Fig. 1) and the transmissibility curve $\widehat{\beta }(\widehat{\delta },\widehat{\rho })$ in Fig. 7—obtained strictly by the numerical methods outlined in Sect. 3—is convincing.

Despite these promising first results, there are many ways in which our approach could be improved. Besides the static patterns, Grenfell et al. discuss phylogenies for different viruses. These phylogenies should be reproducible by our framework, at least to some extent. The intra-host model (Sect. 3.2) generates phylogenies, which could straightforwardly be used for chronic infections. Though one must recall that practically, by introducing a lower load threshold, we effectively trace chronic infections only for 2 years and, in doing so, likely ignore less virulent strains that are tolerated by the host. Acute infections are problematic as well, perhaps even more so as genetic information is not transferred from one to another host in the current intra-host model.

Color-mixing, as utilized for the reconstruction of infection types, is another candidate for improvement. It is sufficient when dealing with three parameters and three infection types. For larger numbers, as required in more detailed settings (cf. Fig. 8), one needs other tools. Although less intuitive, one could keep the finite approximation and modify the linear algebra behind.

More parameters and dimensions would come into play when considering:

(i) a more involved and tunable network model with multiple/intermediate hosts (Read and Keeling 2003; Lewis et al. 2008; Hartlage et al. 2016), including indirect transmissions via vectors, air, water, foot, or smear infection (Ferguson et al. 1999; Ssematimba et al. 2012);
(ii) further parameters (not only $\delta ,\rho$) to be varied by mutation, most importantly infectiousness $\alpha$ (Herfst et al. 2012);
(iii) reassortment (Fuller et al. 2013), possibly as a combination of (i) and (ii);
(iv) more variable durations of infection (Viljoen et al. 2018) (via fine tuned load thresholds $v_0$, fading immunity, etc.), which would allow for more diverse chronic infections (Klenerman and Hill 2005);
(v) virulence (Alizon et al. 2009), possibly via a variable rate $\rho$ of target cell depletion;
(vi) a variable initial viral dose/load (Li and Handel 2014) and the phenomenon of T-cell exhaustion (Wherry et al. 2003; Wherry and Kurachi 2015).

Except for (ii) and parts of (i), (iv), and (vi), the suggested extensions will not be easy to realize within the presented framework. (iv) and (v) would involve elongated time scales, possibly multiple generations of hosts where co-evolution becomes important (Levin 1996; Rehermann 2009). In fact, (iv) has recently been investigated in the limiting case of vanishing thresholds ($v_0=0$) by Viljoen et al. (2018), which resulted in a smaller variety of infection types. When including virulence (v) or other interaction with the host-environment, one must reconsider the fitness definition via $R_0$ (Dieckmann 2002) and possibly also investigate coexisting strains and re-infection of partially immune hosts (Georgieva et al. 2019). Not only to support (iii), the models would have to be more realistic, especially at the intra-host and transmission level, regarding the involved microbiological processes. But also at the inter-host level, one may try to employ dynamical and more structured networks to improve our understanding of viral lifestyles and evolution.

## Footnotes

1 Here we refer to the infectiousness bound $\widehat{\alpha }$, cross-immunity $\chi$, and other intra-host parameters. In our numerical simulations, they are assigned with values from Table 1.

2 Then the simulation runs only take a few hours on a PC.
